# The Emperor Has No Clothes? Searching for Dysregulation in Sepsis

**DOI:** 10.3390/jcm7090247

**Published:** 2018-08-29

**Authors:** Joe Alcock

**Affiliations:** Department of Emergency Medicine, University of New Mexico, Albuquerque, NM 87131, USA; joalcock@salud.unm.edu; Tel.: +1-505-272-5062

**Keywords:** sepsis, dysregulation, organ dysfunction, early goal-directed therapy, adaptation, evolution

## Abstract

The core conception of sepsis—that it is a dysregulated state—is a powerful and durable idea that has inspired decades of research. But is it true that the body’s response to sepsis is dysregulated? To answer that question, this review surveyed the history of trials of experimental sepsis treatments targeting the host response. Sepsis survival is not improved by blocking one or many immune pathways. Similarly, sepsis is resistant to treatment by normalizing one or many physiologic parameters simultaneously. The vast majority of interventions are either ineffective or harmful. With this track record of failure, it is time to consider the alternative hypothesis—regulation instead of dysregulation—and the possibility that sepsis traits are often functional, and that some physiologic alterations in sepsis do more good than harm, while others are neutral. This review discusses the implications of this perspective for the future of sepsis research.

## 1. Introduction

### “We Have Met the Enemy and He Is Us”—Walt Kelly

For decades, sepsis research has been motivated by the idea of a dangerous overreaction of the immune system. Lewis Thomas wrote about microbes in sepsis: “It is our response to their presence that makes the disease. Our arsenals for fighting off bacteria are so powerful … that we are more in danger from them than the invaders” [1].

In the Third International Consensus Definition of Sepsis and Septic Shock, Thomas’s viewpoint is built into the definition of sepsis: “a life-threatening organ dysfunction caused by a dysregulated host response to infection” [2]. A dominant view of sepsis is that injury and death result from a cascade of inflammatory products that damage the microvasculature and cause multiorgan failure [3]. Thus, the hypothesis that sepsis is a dysregulated response implies that attenuating the immune response or blocking some critical immune pathway will improve patient outcomes.

Physicians use the infected patient, not the infecting agent, to define and detect sepsis at its earliest stages. Instead of measuring the causative pathogen(s), we use proxy measures. Fever, low blood pressure, increased respiratory rate, and somnolence alert us that there is a problem. Nearly every physiologic change of sepsis, observable during physical exam or measured in routine tests or at the molecular level, has been viewed as a target for medical intervention. The assumption is that each sepsis-associated finding participates in the pathological process.

Recently, Alverdy and Krezalek wrote of the US National Institutes of Health, by far the largest funder of sepsis research: “with no exception, every funded grant based on the immunocentric theory of sepsis promises that … blockade of a pathway or molecule will improve the outcome of human sepsis research. In order for the immunocentric view to prevail, the cause of death from sepsis must be believed to be due to the response itself and not to the inciting pathogen” [4].

Despite a well-funded effort to uncover the underlying mechanisms of septic shock, effective new therapies have proven to be stubbornly hard to find. Multiple randomized controlled trials have tested new agents with the aim of improving sepsis survival. Only one immune-modifying drug, recombinant activated protein C, passed through phase 3 clinical trials, gaining Food and Drug Administration (FDA) approval [5]. Despite this temporary success, later definitive trials of recombinant activated protein C showed it to be ineffective [6], and it was taken off the market in 2011. 

These failures notwithstanding, many physicians, researchers, and grant agencies hold firm to the idea that the human body’s response itself is a problem in sepsis. Many authors have pointed to the lack of progress in treating sepsis and have offered various solutions. These solutions include more careful patient selection [7], stratifying patients by severity of disease [8], and more careful selection of targets for intervention [9]. The timing and intensity of interventions have also been cited as reasons for the failure of certain trials and are the subject of ongoing debate [10]. Another potential solution comes from the recognition that sepsis involves different immune states. Excessive activation of immunity can be followed or preceded by immune paralysis or exhaustion [11,12], necessitating different interventions depending on the time course of the disease. 

While the search for new ways to protect the host from the immune response is ongoing, the track record of sepsis treatments should prompt a reevaluation of the central premise of sepsis, that it is a dysregulated host response to infection. A more fruitful solution might be to ask: where does dysregulation exist, if at all, in sepsis? 

## 2. Dysregulation in Sepsis versus Function

The body of knowledge accumulated in the last half-century can be thought of as a test of the dysregulation hypothesis—that the body’s response causes more harm than good in sepsis. Unlike the explanations for the lack of progress offered by others [13], the assumption that sepsis is dysregulated has gone mostly unchallenged [4]. This review will highlight the history of sepsis trials to see whether those cumulative results support a key prediction of the dysregulation hypothesis: if sepsis involves dysregulation, then interventions targeting dysregulated pathways are predicted to improve outcomes. 

An alternative hypothesis is that some sepsis phenomena are functional, regulated traits. Clinical trials offer tests of the potential function of phenotypes in sepsis. In molecular biology, uncovering the function of a gene or protein is often accomplished by using knockout mice, in which a genetic coding region is altered or deleted. By comparing those phenotypes to the phenotypes of unaltered animals, a gene’s or protein’s function can be inferred. Immunomodulatory drugs are not the same as knockouts, but they can have similar effects by blocking downstream effects of gene expression. Like knockout models, many sepsis trials shed light on the question of the function of underlying traits. If those traits are functional, then blocking them should fail to improve outcomes, and might do more harm than good.

## 3. The Failure of Immunomodulators in Sepsis

The treatment of septic shock is marked by pervasive and ongoing controversy involving nearly all elements of sepsis care, with the possible exception of antibiotics. At the core of sepsis treatment is the notion that sepsis is a dysregulated harmful response to an infectious challenge, with an immune response that is out of control [14,15]. However, if it is true that the immune response in sepsis is usually dysfunctional, then it follows that interventions that block endogenous responses to sepsis should provide a survival benefit. In 2002, Eichacker identified over 20 randomized controlled trials of anti-inflammatory agents used in sepsis. Despite promising results using experimental animal models, these agents uniformly failed in phase 3 human clinical trials. By 2014, the tally of failed trials was higher. Marshall identified over 100 randomized clinical trials of immune modulating agents in sepsis. None has led to a durable new treatment [16] (Table 1).

Medical reversal is a problem that afflicts biomedical science generally [17], but it seems particularly an issue for clinical trials of sepsis therapies. A *Nature* review published in 2002 outlined four interventions targeting the host response in sepsis supported by human trials [14]. Results of each of these four treatment strategies, involving low-dose corticosteroids, optimization of hemodynamic function [18], intensive glucose control [19], and activated protein C [5], all failed to be replicated in subsequent trials, as we will describe below. 

The next sections will review the recent history of sepsis interventions and survey the record of trials testing key molecular and physiologic targets to see if evidence exists for dysregulation.

### 3.1. Lipopolysaccharide/TLR4 Pathway

In 1985, Beutler et al. showed that mortality in mice could be reversed by blocking the host response to lipopolysaccharide (LPS) [20]. This apparent success spurred an explosion of research into immunomodulatory agents aimed at improving outcomes in human sepsis. Antibodies to LPS were among the first tested. Despite promising preclinical results, multiple trials of antibodies to LPS failed in humans [7]. Following those failures, additional efforts were undertaken to block the interaction between LPS and toll-like receptor 4 (TLR4). In 2013, the results of a clinical trial of eritoran tetrasodium, a second-generation anti-LPS/TLR4 agent, were published. The ACCESS trial was a multicenter randomized controlled trial of eritoran involving 1961 sepsis patients in three intensive care units (ICUs). Patients with sepsis and evidence of organ dysfunction were randomized in a 2:1 ratio, with 1304 assigned to eritoran and 657 to placebo [21]. Unfortunately, the TLR4 blocker eritoran did not reduce all-cause mortality at 28 days or at 1 year. The drug joined a growing list of failed drugs for sepsis.

These results together do not support the idea that the LPS/TLR4 pathway is dysregulated in sepsis. Instead, having a functional capacity to respond to LPS with TLR4—the sensing and effector pathway triggered by invasive gram-negative bacteria—is likely to be a functional trait in sepsis.

### 3.2. Recombinant Activated Protein C

Acquired deficiency of protein C was shown to be a predictor of mortality in patients with sepsis, reviewed in [22]. A recombinant form of activated protein C was shown in animal and human studies to have anti-inflammatory and anticoagulant properties. Since inflammation was thought to be out of control in sepsis, investigators reasoned that an inhibitor of inflammation and clotting would potentially provide a treatment. The PROWESS trial, published in 2001, reported a survival benefit in patients with sepsis treated with recombinant activated protein C [5]. On the basis of those results, the FDA approved recombinant activated protein C (Xigris^®^, Eli Lilly, Indianapolis, IN, USA) as the only immune-modulating agent ever approved specifically for sepsis. The results and methodology of the PROWESS trial received criticism, mainly for a midstream change in protocol [23]. Reinforcing the skepticism over Xigris, subsequent randomized controlled trials failed to show benefit. The F1K-MC-EVBP trial of activated protein C in pediatric sepsis was terminated early for futility. A study involving patients with a low risk of death, the ADDRESS trial, also failed to show benefit [24]. Because of this increasing uncertainty, the European Medicines Agency requested another trial to confirm the results of PROWESS in patients with severe shock. The resulting PROWESS-Shock trial enrolled patients with severe sepsis. In contradiction to the original PROWESS results, patients given recombinant activated protein C had no survival benefit in PROWESS-Shock [6]. A subsequent meta-analysis confirmed this absence of benefit and an increase in adverse effects, mostly bleeding [25]. Ten years after the original PROWESS trial, on October 25, 2011, the FDA recommended that Xigris be withdrawn from the market.

Antithrombin III, an endogenous anticoagulant like activated protein C, has been tested to determine its effect on sepsis survival. As with recombinant activated protein C, provision of high-dose antithrombin III failed to improve sepsis survival and resulted in increased bleeding events [26]. These studies failed to support the hypothesis that the coagulation cascade is dysregulated, on average, in sepsis. The concept of deficiency of activated protein C may be an artifact of mistaken assumptions of dysregulation. These studies provide a clue that decreased protein C activity, and increased activity of the coagulation system, may be functional in sepsis. One proposed hypothesis is that increased activity of the coagulation cascade that occurs during severe infections may promote the trapping and clearance of pathogens [27].

### 3.3. Statins

Statins inhibit the HMG-CoA reductase enzyme, thereby reducing low-density lipoprotein (LDL) cholesterol. Because statins also have anti-inflammatory properties independent of their lipid-lowering effects, they have generated interest as a treatment for sepsis. After promising observational trials, the largest randomized controlled trial (SAILS trial) was recently undertaken to test the effect of rosuvastatin in patients with sepsis-induced lung injury. The SAILS trial failed to show a survival benefit of rosuvastatin and was stopped early for futility [28].

These results do not support the idea that HMG-CoA reductase is dysregulated in sepsis. The available evidence suggests instead that immune modulation by MHG-CoA reductase is neutral or potentially beneficial in sepsis. 

### 3.4. Corticosteroids

Recently the ADRENAL trial was designed to show whether glucocorticoids improve survival in sepsis. The ADRENAL study was a large well-designed trial that enrolled over 3600 patients with septic shock from 69 medical-surgical ICUs in five countries (Australia, New Zealand, Denmark, Saudi Arabia, UK). It found no survival benefit for hydrocortisone in sepsis. Published after the ADRENAL trial, the APROCCHSS trial studied 1241 patients with septic shock and reported a survival benefit for those receiving hydrocortisone and fludrocortisone [29]. In that study, patients with septic shock treated with the combination had a slight mortality benefit. 

Notably, the APROCCHSS study found no survival benefit at day 28. The fact that the reported benefit appears, disappears, and appears again depending on the time point in APROCCHSS suggests that a survival benefit from giving steroids is minimal if it does exist. APROCCHSS has a fragility index of 3 for the outcome of mortality, meaning that the statistical significance in this study is not robust and may be a result of chance alone. Meanwhile, the largest and best designed study, ADRENAL, showed no benefit. Taken together, these studies do not support the idea of pathological adrenal insufficiency in sepsis, nor do they support a view of sepsis as a dysregulated inflammatory condition that is remedied by exogenous corticosteroids. 

One of the most compelling arguments in favor of corticosteroids has been the well-documented “reversal of shock”—less requirement for vasopressors, fewer days on the ventilator in the ICU. It is notable, however, that despite “reversing shock,” glucocorticoids have not, as shown in meta-analysis, improved mortality in sepsis [30]. Furthermore, as we shall see in the remaining sections, attempts to reverse shock by other means, e.g., by treating hypotension and hypoperfusion with intravenous (IV) fluids, have a poor track record of improving sepsis outcomes. 

## 4. Normalization of Sepsis Physiology

Many recent advances in critical care medicine have highlighted the problem of overtreatment and have resulted in physicians often doing less to patients, not more [31]; for example, ventilating patients with “normal” lung volumes as in healthy patients, aggressive treatment of anemia with blood transfusion, and intensive glucose control have all recently been shown to be harmful in septic ICU patients [31]. Here we review some of the most recent trials involving normalization of physiology in sepsis to see whether they improved or worsened patient outcomes.

### 4.1. Fever

Several large observational trials have shown that among patients with sepsis or severe infection, the absence of fever is associated with a greater chance of death. A study of 2225 patients with sepsis in Sweden showed that increased body temperature was associated with improved survival, and that higher fevers were more protective [32]. In addition, large observational trials of ICU patients in Denmark, New Zealand, Australia the UK, and the US have shown similar improved survival in those with fever [33,34,35]. Lack of fever or low body temperature heralds a poor prognosis in sepsis.

Randomized controlled trials of interventions targeting fever have shown similar results to observational trials. Using active methods to cool body temperature in the recently published CASS trial tended to increase mortality in sepsis [36]. The CASS trial authors wrote: “After recruitment of 436 of the planned 560 participants, the trial was terminated for futility (220 [50%] randomly allocated to hypothermia and 216 [50%] to routine thermal management, e.g., with antipyretics)” [36]. Further, “In the hypothermia group, 96 (44.2%) of 217 died within 30 days versus 77 (35.8%) of 215 in the routine thermal management group (difference 8.4% [95% CI) −0·8 to 17.6]; relative risk 1.2 [1.0–1.6]; *p* = 0.07]).” The CASS trial was stopped early because of concern that cooling was harmful.

Two randomized controlled trials posed the question of whether antipyretic medications were helpful in sepsis. Bernard et al., in 1997, published the results of a randomized, double-blind, placebo-controlled trial of intravenous ibuprofen versus placebo in 455 patients who had sepsis [37]. Ibuprofen did not improve survival in that study. The effect of acetaminophen, the most commonly used antipyretic, on survival was the subject of a randomized controlled trial by Young et al.; the HEAT trial showed no benefit to sepsis mortality from acetaminophen (paracetamol) [38].

Is fever a harmful state of dysregulation in sepsis? These trials do not support that hypothesis. The observational studies cited above signal instead that having a fever is protective for patients with sepsis. Increased mortality suggested by the CASS trial casts doubt on the wisdom of fever reduction in the emergency department (ED). Taken together, these studies support the concept that elevated temperature is a regulated adaptive reaction to infection and sepsis.

### 4.2. Elevated Blood Sugar

In 2001, the *New England Journal of Medicine* published a paper by van den Berghe et al. that showed improved survival in critically ill patients treated with intensive blood sugar control using insulin. This study led to an increase in “tight glycemic control” seeking to normalize hyperglycemia in the intensive care unit. In 2009, another paper refuted the results of the original trial. The NICE SUGAR study enrolled 6000 critically ill patients, randomizing 3000 of them to tight glycemic control. The investigators found that mortality was significantly higher in the tight glycemic control treatment arm (27.5% vs. 24.9%).

Because the NICE SUGAR study did not involve children, Agus et al. [39] performed a randomized controlled trial of aggressive insulin treatment of hyperglycemia in pediatric patients in intensive care. This was not a study of pediatric sepsis, specifically, but many enrolled patients had infection and sepsis. Agus et al. [39] enrolled 713 critically ill children and randomly assigned them to a lower-target blood sugar group who received more insulin and a higher-target group who received less insulin. The main outcome measure, ICU-free days up to day 28, was similar in both groups. Mortality was also similar. This study was stopped early, because the interim analysis determined a low likelihood of benefit from giving more insulin and a high risk of harm. Among enrolled children in this study, those receiving more insulin had increased health care–associated infections (12 of 349 patients (3.4%) vs. 4 of 349 (1.1%), *p* = 0.04) in the group receiving less insulin.

The NICE SUGAR and Agus et al. studies do not support the concept that acquired insulin resistance is dysregulated in critically ill adults or children, although a study tailored to sepsis would be helpful. It may be time to consider this trait as neutral or possibly beneficial in critically ill and septic patients.

### 4.3. Sepsis Bundles and Early Goal Directed Therapy

**Figure 1:**
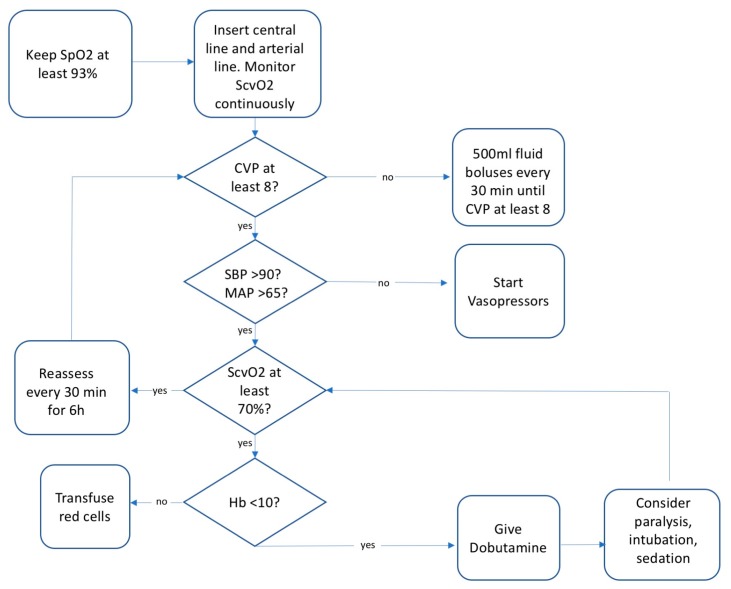
Early goal-directed therapy (EGDT) algorithm of care that was randomized to the treatment of a group of patients with septic shock in the ProMISe trial. For the 1251 patients in this large trial, there was no difference in 90-day mortality, indicating that EGDT does not improve outcomes in sepsis. SpO_2_, oxygen saturation; CVP, central venous pressure; SBP, systolic blood pressure; SCVO_2_, central venous oxygen saturation; Hb, hemoglobin.

Early goal-directed therapy (EGDT) for sepsis was an idea that gained relevance with the influential Rivers trial [1], a single-center randomized controlled trial in which a variety of physiologic parameters were treated simultaneously, aiming to bring them closer to normal values in an effort to restore homeostasis. EGDT prescribed multiple simultaneous interventions in sepsis: blood transfusions for anemia, dobutamine for cardiac output, normalizing central venous pressure, and maximizing oxygen delivery (Figure 1). Unfortunately, many of the interventions (including dobutamine) were shown to be ineffective when tested individually in later trials and were deleted from the bundle. Still, the concept of early goal-directed therapy itself was not tested until three randomized controlled trials were conducted, starting in 2008 and published in 2014–2015. ProCESS, ARISE, and ProMISe were large-scale randomized controlled trials of EGDT independently conducted on three continents [40,41,42]. In each study, EGDT failed to improve survival compared to usual care. The collective failure of these trials casts doubt on the notion of maladaptive dysregulation in sepsis. It also raises questions about the assumption of inadequate tissue oxygen and perfusion in septic shock, an idea that itself has been criticized [43].

EGDT was once enshrined in sepsis treatment bundles championed by the Surviving Sepsis Campaign (SSC), which started as a marketing arm of Eli Lilly [23]. Not coincidentally, Eli Lilly was the maker of recombinant activated protein C, the medication that was included in early Surviving Sepsis guidelines and later withdrawn from the market. The Surviving Sepsis Campaign is no longer affiliated with the drug manufacturer. Since the original Rivers trial [18], sepsis bundles advocated by SSC have contained useless elements—measuring central venous oxygen saturation (SVCO_2_), maintaining central venous pressure (CVP) of 8 mmHg—and demonstrably harmful ones—Xigris, dobutamine, and high transfusion targets. The 2017 Surviving Sepsis treatment bundle has since deemphasized EGDT, but it still prioritizes quick administration of antibiotics, on which there is broad agreement, and provision of fluids, controversially within one hour [44].

Acceptance of the dysregulation hypothesis explains the eagerness with which EGDT was adopted. If the body’s response is the problem, then reversing sepsis physiology by normalizing multiple parameters simultaneously is expected to make patients better, not worse. However, in the nearly two decades since the influential Rivers trial, EGDT has been shown to be ineffective, and the current SSC bundles rest on a thin evidence base, reviewed in [43].

We will examine the quality of that evidence and the proposal that sepsis involves dysregulated hypoperfusion in the next section.

### 4.4. Fluid Therapy

Fluid therapy is a mainstay of sepsis treatment, advocated by the Surviving Sepsis Campaign and aimed at normalizing blood pressure and increasing tissue perfusion. Despite much interest in optimal fluid therapy, only two randomized controlled trials of fluid boluses versus no boluses have been performed in patients with sepsis. These two trials were conducted in Africa, in countries where standard of care does not include intravenous crystalloid fluid therapy. Maitland and colleagues randomized children with sepsis in three African countries to receive intravenous fluid boluses or usual care [45]. In that study, the FEAST trial, pediatric patients with sepsis who were randomized to fluid boluses had higher mortality compared to pediatric patients with hypotensive shock who did not receive intravenous fluids [45].

A similar study in adults with septic shock was performed by Andrews et al. [46]. Adults with sepsis (*n* = 209) presenting to an emergency department in Zambia who were randomized to receive intravenous fluids, vasopressors, and blood transfusion had significantly higher mortality in hospital compared with usual care (48.1% vs. 33.0%, respectively) [46]. In both trials, one involving children and the other adults in African countries, more patients died when they received IV fluids. In other words, the care that is assumed to be life-saving in emergency departments and intensive care units in the developed world harmed study patients in these developing countries.

Giving no fluids has not been thought to be an option in human trials of sepsis in developed countries. However, conservative or limited fluid strategies have been tested. Silversides et al. showed that a conservative fluid strategy was associated with better survival in sepsis [47]. At least for the populations for which we have randomized controlled trials, these studies do not support the idea that we improve dysregulated tissue perfusion by infusing IV crystalloid in sepsis.

Marik and Bellomo have argued that the problem with IV fluids in sepsis includes damage to the endothelial glycocalyx and harmful tissue edema [43]. They advocated using vasopressors instead to treat the hemodynamic abnormalities of sepsis. Maitland, the principal investigator in the FEAST trial, provocatively suggested that hemodynamic abnormalities such as hypotension have a defensive function, implying they should be left alone in some cases. Maitland was quoted in a recent *Lancet* article as saying, “Our theory is that the shock response in severe febrile illness is a defense mechanism, and bringing [children] out of this too soon with a fluid bolus can be counterproductive” [48].

### 4.5. Organ Failure

Mervyn Singer has pointed out the paradox of “clinical and biochemical organ failure in sepsis yet minimal cell death” and proposed that organ failure represents a state of hibernation in the face of overwhelming inflammation that helps to promote survival [49]. Any potential benefit from organ failure in sepsis must be considered in the context of observational trial data linking the amount and degree of organ failure with mortality [50]. Septic acute kidney injury follows a similar pattern of increased mortality with organ dysfunction [51]. However, animal models of septic acute kidney injury suggest that the marked reduction in glomerular filtration is not accompanied by tissue injury, significant cell death, or histological inflammation [52]. In an ovine model of sepsis, blood flow to the kidney was preserved and there was no evidence of inflammation, and no tubular cell necrosis or blood vessel damage [53]. Recovering from acute kidney injury returned survivors to pre-sepsis levels of function, suggesting that there was no long-term damage. Maiden et al. concluded from these findings that septic acute kidney injury is a functional response; in other words, an adaptation [53].

Studies examining the utility of renal replacement therapy have not shown consistent benefit for sepsis patients, although a multicenter randomized trial is ongoing. A recent review comparing the survival of patients randomized to receive early versus late initiation of renal replacement concluded that there was no clear benefit of early renal replacement in randomized controlled trials [54]. This lack of benefit, along with the absence of cellular injury in renal failure, provides preliminary but not conclusive evidence that septic reduction in the glomerular filtration rate might be a regulated trait, not a dysregulated one.

### 4.6. Interventions with No Verdict Yet

Not every potential molecular pathway of sepsis has been studied, and one merits special discussion. Current interest in vitamin C, thiamine, and steroids increased after an observational trial of those agents combined suggested a robust survival benefit [55]. Although treatment bundles and hydrocortisone have checkered track records in sepsis, as discussed above, vitamin C has generated considerable interest as an antioxidant and immune-modifying agent. Additionally, endogenous levels of vitamin C in sepsis are lower than those in healthy individuals, potentially representing a deficiency state amenable to treatment [56]. Correcting other supposed deficiency syndromes of sepsis, e.g., of activated protein C or calcium, have not improved survival [6,57]. Similarly, trials that randomized critically ill patients to vitamin C have not yet shown a survival benefit, reviewed in [56]. Despite these headwinds, an ongoing randomized controlled trial involving vitamin C, thiamine, and steroids is in progress. Vitamin C has another potential benefit that does not rely on anti-inflammatory or antioxidant effects or remedying a deficiency: direct and synergistic antibacterial effects [58]. The dose-dependent inhibition of human pathogens by vitamin C might be beneficial during infection.

## 5. Regulation and Adaptation in Sepsis

The treatment of sepsis has been turbulent in recent decades, with some short-lived moves forward often followed by medical reversal. Over time, many published reviews of the state of sepsis treatments have followed a repetitive pattern. Authors catalogue previous failures, bemoan the absence of progress, and point to upcoming trials of promising new therapies. Later reviews resemble their predecessors, except the list of failed treatments now contains the once-promising agents.

Over the same time, hospital mortality attributed to sepsis has decreased, in one study from 24.1% to 14.8% [59], a trend that some attribute to the Surviving Sepsis Campaign recommendations that now rest on a thin evidence base. The decreased mortality may be plausibly attributed to increased awareness and screening identifying more patients as having sepsis, consistent with the nearly threefold increase in hospital admissions for sepsis during the same period [59]. Improved outcomes were not likely to have resulted from interventions aimed at restraining a dysregulated immune response.

The preponderance of evidence indicates that sepsis survival is not improved by blocking one or many immune pathways. Similarly, improvements in sepsis mortality are resistant to modification by normalizing one or many physiologic parameters simultaneously. The vast majority of interventions are either ineffective or harmful. As a predictive heuristic, the dysregulation hypothesis has a repeated track record of failure, and an even more remarkable durability. Now, given the choice between dysregulation and regulation, it may be time to consider the alternative hypothesis—regulation instead of dysregulation—and seriously consider the possibility that some sepsis phenotypes represent regulated functional responses.

Our ancestors faced infectious challenges since the first multicellular organism evolved. Some disease-associated findings, such as fever, are hypothesized to occur because of natural selection acting on our vertebrate ancestors [47]. Organisms with effective host defenses against overwhelming infection were more likely to survive and reproduce, leaving extant organisms with a genetic toolbox of defenses. Adaptation by natural selection provides an explanation for normal human physiology and constitutes a framework to identify potential functional responses, or “defenses,” during disease [60]. It is outside the scope of this review to weigh the merits of all adaptive proposals for various sepsis traits. However, adaptive hypotheses in medicine, as in biology, must meet specific conditions, as described in 1966 by Williams [61]; these should include a biologically plausible mechanism and should be prospectively tested against nonadaptive alternative hypotheses [62].

Because pathogens evolve too, the toolbox of host defenses is never perfect. Lethal competition between humans and pathogens can resemble a co-evolutionary arms race in which each side gains temporary advantage over the other [63]. This means that we will never see an organism, or a patient, perfectly adapted to resist infection. Other reasons for nonoptimal sepsis traits include aging, prior injury, environmental toxins, energetic or biological constraints, environmental changes for which we are not evolved (like the intensive care unit), and immune trade-offs that protect us from one pathogen while leaving us vulnerable to another. For these reasons, an individual’s response in sepsis may indeed be pathological in some instances. In other cases, the host may gamble and lose on an immune strategy that pays off on average. This concept has been termed “immune brinksmanship,” a high-stakes contest in which hosts use defenses that preferentially harm invasive pathogens but can also injure the host [64].

Is it true in sepsis, as Lewis Thomas wrote, that “our arsenals for fighting off bacteria are so powerful … that we are more in danger from them than the invaders”? Apparent self-harm from the immune system has appeared paradoxically excessive to students of sepsis for decades [37]. More recent work involving myeloid-derived suppressor cells demonstrates the immune system’s potential for harm in sepsis, and also reveals functional regulation that is required for good outcomes [65]. The cumulative record of human sepsis trials suggests that we may be better off with our immune arsenals than without them. If this supposition is true, then breakthroughs in sepsis are less likely to come from inhibiting the host response and more from targeting the microorganisms that are ultimately responsible for sepsis and septic shock. Timely antibiotics and source control of infection are mainstays of sepsis treatment [10]. Another promising area of research is how the microbiome might protect some patients and predispose others to sepsis [4]. On the host side, it is no accident that many advances in critical care have come from intervening less aggressively [31]. If many or most host responses in sepsis are functional, that trend is likely to continue. Alternatively, augmenting select responses is an idea that is gaining traction [66]. Deciding whether to intervene, and in what direction, remains a key challenge. Correctly identifying function versus dysfunction and regulation versus dysregulation will be a step in the right direction.

## Figures and Tables

**Table 1:** Targets of treatment in sepsis, adapted from [16].

Dysregulated Pathway	Sepsis Treatment
**Lipopolysaccharide (LPS; endotoxin)**	
	Anti-LPS human monoclonal antibody HA-1 Anti-LPS E5 murine monoclonal IgM antibody
	Enterobacterial common antigen
	Toll-like receptor 4 antagonists
	Eritoran
	TAK-242 (resatorvid)
	Anti-CD14
	Taurolidine
	Alkaline phosphatase
	Polymyxin B
	Lipid emulsion
**Tumor necrosis factor (TNF)**	Monoclonal or polyclonal antibodies
	Soluble receptor constructs
**Interleukin-1 (IL-1)**	Recombinant IL-1 receptor antagonist
	Small-molecule inhibitors
**Platelet activating factor (PAF)**	PAF acetylhydrolase
	Ibuprofen
**Eicosanoids**	Phospholipase A2 inhibitor
	NO synthase inhibitor l-NNMA
**Hypercoagulability**	Methylene blue
	Activated protein C
	Tissue factor pathway inhibitor
	Antithrombin III
	Anti-tissue factor antibody
	Thrombomodulin
**Immune suppression**	Intravenous (IV) immunoglobulin
	Granulocyte Colony Stimulating Factor and Granulocyte/Macrophage Colony Stimulating Factor
**Adrenal insufficiency**	Corticosteroids
	Statins
**Organ failure**	Extracorporeal hemoperfusion
**Inadequate perfusion**	IV fluid bolus
	Dobutamine
**Insulin resistance, metabolism**	Intensive insulin
